# Commentary: Can Inner Experience Be Apprehended in High Fidelity? Examining Brain Activation and Experience from Multiple Perspectives

**DOI:** 10.3389/fpsyg.2018.01243

**Published:** 2018-08-03

**Authors:** Stuart J. McKelvie

**Affiliations:** Department of Psychology, Bishop's University, Sherbrooke, QC, Canada

**Keywords:** consciousness, pristine inner experience, theory, autobiographical memory, flashbulb memory, imagery vividness

I agree with Hurlburt et al. (2017a) that conscious experience is a universal human characteristic, and worthy of scientific study. Science involves describing (measuring), classifying, and testing theories about functions by examining non-causal and causal relationships with other variables. Has this been accomplished for pristine inner experience?

For *description*, Descriptive Experience Sampling (DES) is a disciplined iterative procedure that requires careful training (Hurlburt, 2009; Hurlburt and Heavey, 2015; Lapping-Carr and Heavey, 2017). Unlike “directly apprehended” inner experience (Hurlburt et al., 2017a, p. 3), “DES-apprehended experience” (Hurlburt et al., 2017b, p. 3) is not pristine, but *aspires* to obtain a faithful description. As evidence for the fidelity of DES reports, Hurlburt et al. (2017a) summarize evidence that brain activation differ across inner experiences.

Here, “apprehend” is used in two ways: having a natural pristine experience and later reporting it. This is potentially confusing. Discussing DES, Hurlburt et al. (2017b, p. 3) state that they used apprehension to imply “proximity, involvement, intervention,” which recognizes that the description cannot perfectly represent the original experience. Perhaps they should avoid “apprehend” when referring to the original experience.

For *classification*, Heavey and Hurlburt (2008) conducted a taxonomic study by accumulating idiographic DES reports and aggregating them to provide nomothetic information (see Hurlburt and Akhter, 2006; Hurlburt and Heavey, 2006, p. 209).The five most frequent categories were inner speech, inner seeing, unsymbolized thinking, feelings, and sensory awareness (see also Hurlburt and Akhter, 2008; Heavey et al., 2012; Hurlburt et al., 2013). Inner seeing was slightly more frequent than the others. People also differed in which category was most prominent.

For *relationships*, there were no sex differences, but psychological distress was negatively correlated with inner speech and positively correlated with unsymbolized thinking. Furthermore, Hurlburt et al. (2002) found that people who had high or low speech rates differed on 5 out of 11 kinds of pristine inner experience. Finally, Hurlburt and Heavey (2006, Chapter 14) offer speculations about individual differences in patterns of inner experience and relationships to other variables.

For *theory*, Hurlburt and Akhter (2008) propose a division of labor between people who describe pristine inner experiences and people who theorize about them, because investigators of introspection tangled method and explanation in their search for the elements underlying experience (Hurlburt et al., 2017a). Thus, Hurlburt et al. (2017b, p.2) “bracketed away” theoretical questions, particularly how pristine inner experiences are caused and whether they have effects. Given their careful efforts to obtain faithful records of inner experience, Hurlburt and colleagues should test theoretical issues. Others have used their measurement instruments in this way.

However, Hurlburt and colleagues do not deny “causative significance” of pristine inner experience (Hurlburt et al., 2017b). Indeed, Hurlburt and Heavey (2006) speculative relationships are “based on the belief that people's patterns of inner experience could influence (and be influenced by) how they encounter the world” (p. 233).

## Autobiographical memory and imagery

One of Hurlburt et al.'s (2017a) DES reports contains a detailed description of visual memories for a personal event (p. 3, Sample 5.1). This provokes three theoretical questions with implications for pristine inner experience. Are autobiographical memories accurate? Can re-experiencing events have consequences? Can vivid visual imagery have consequences?

All personal recollections involve reconstruction, which leaves open the possibility of recall errors (Holland and Kensinger, 2010). Specifically, for natural autobiographical memory, Barclay and Wellman (1986) obtained records of personal events over four months. Subsequently, they found evidence of both accurate and inaccurate memory. Similarly, for flashbulb memory, where people report powerful remembering of where they were and what they were doing when an important event occurred (Hirst and Phelps, 2016), there is evidence of accuracy and inaccuracy (Schmolck et al., 2000; Berntsen and Thomsen, 2005; Hirst et al., 2015). Pristine inner experiences that involve such memories may also be accurate and inaccurate.

Experiencing autobiographical memories can have beneficial effects, such as maintaining a sense of self (Holland and Kensinger, 2010; Hallford and Mellor, 2016; Vannucci et al., 2016) and bolstering self-esteem. Based on such evidence, some of which is experimental, reminiscing has been employed as a therapeutic intervention (Hallford and Mellor, 2016). When pristine inner experiences involve autobiographical memories, there may show beneficial effects.

In Hurlburt et al.'s (2017a) example, the visual images seem to be clear, detailed, and lifelike, which are the characteristics of vivid imagery (Marks, 1972, 1999). Two researchers (Richardson, 1984, p. 100; Marks, 1999) claim that vivid imagery has effects. However, although extensive experimental evidence supports causation for visual imagery in general (e.g., Smith and Over, 1987; Grouios, 1992; Ironsmith and Lutz, 1996), studies of vividness of visual imagery are nonexperimental. Vividness has been examined from ratings of materials and from self-reports, with evidence of various positive relationships (McKelvie, 1995; D'Angiulli et al., 2013; Runge et al., 2017).

To examine causation, people should be instructed to generate visual images that vary in vividness. Similarly, inner seeing and other pristine inner experiences should also be experimentally manipulated. This might be challenging, because these experiences occur naturally (Hurlburt et al., 2017a) and may not be the same as those that are generated consciously. Moreover, it may be more difficult to manipulate some kinds of pristine inner experience (e.g., unsymbolized thinking, sensory awareness) than others (e.g., inner speech, inner seeing, feeling).

## Conclusion

Hurlburt and colleagues have developed an interesting method to faithfully capture pristine inner experiences in natural settings. For DES reports, there are no reliability data, which are required to study individual differences. However, preliminary work has classified these experiences, and demonstrated their relationships with other variables.

The most important theoretical issue is whether pristine inner experiences play any causal role. Research shows that reminiscing has benefits, and that vividness of visual imagery is positively related to objective performance. This suggests that pristine inner experience, particularly inner seeing, may have effects. Experimentation faces serious methodological challenges, but research with DES reports should examine suggested relationships among them and with behavior in a nomological network (Hurlburt and Heavey, 2006, pp. 58, 237). This would also provide evidence of their construct validity.

## Author contributions

The author confirms being the sole contributor of this work and approved it for publication.

### Conflict of interest statement

The author declares that the research was conducted in the absence of any commercial or financial relationships that could be construed as a potential conflict of interest.
